# CD38 Structure-Based Inhibitor Design Using the *N*1-Cyclic Inosine 5′-Diphosphate Ribose Template

**DOI:** 10.1371/journal.pone.0066247

**Published:** 2013-06-19

**Authors:** Christelle Moreau, Qun Liu, Richard Graeff, Gerd K. Wagner, Mark P. Thomas, Joanna M. Swarbrick, Satoshi Shuto, Hon Cheung Lee, Quan Hao, Barry V. L. Potter

**Affiliations:** 1 Wolfson Laboratory of Medicinal Chemistry, Department of Pharmacy and Pharmacology, University of Bath, Bath, United Kingdom; 2 Macromolar Diffraction Facility, Cornell High Energy Synchrotron Source, Cornell University, Ithaca, New York, United States of America; 3 Department of Pharmacology, University of Minnesota, Minneapolis, Minnesota, United States of America; 4 Faculty of Pharmaceutical Sciences, Hokkaido University, Sapporo, Japan; 5 Department of Physiology, University of Hong Kong, Hong Kong, China; Russian Academy of Sciences, Institute for Biological Instrumentation, Russian Federation

## Abstract

Few inhibitors exist for CD38, a multifunctional enzyme catalyzing the formation and metabolism of the Ca^2+^-mobilizing second messenger cyclic adenosine 5′-diphosphoribose (cADPR). Synthetic, non-hydrolyzable ligands can facilitate structure-based inhibitor design. Molecular docking was used to reproduce the crystallographic binding mode of cyclic inosine 5′-diphosphoribose (*N*1-cIDPR) with CD38, revealing an exploitable pocket and predicting the potential to introduce an extra hydrogen bond interaction with Asp-155. The purine C-8 position of *N*1-cIDPR (IC_50_ 276 µM) was extended with an amino or diaminobutane group and the 8-modified compounds were evaluated against CD38-catalyzed cADPR hydrolysis. Crystallography of an 8-amino *N*1-cIDPR:CD38 complex confirmed the predicted interaction with Asp-155, together with a second H-bond from a realigned Glu-146, rationalizing the improved inhibition (IC_50_ 56 µM). Crystallography of a complex of cyclic ADP-carbocyclic ribose (cADPcR, IC_50_ 129 µM) with CD38 illustrated that Glu-146 hydrogen bonds with the ligand *N*6-amino group. Both 8-amino *N*1-cIDPR and cADPcR bind deep in the active site reaching the catalytic residue Glu-226, and mimicking the likely location of cADPR during catalysis. Substantial overlap of the *N*1-cIDPR “northern” ribose monophosphate and the cADPcR carbocyclic ribose monophosphate regions suggests that this area is crucial for inhibitor design, leading to a new compound series of *N*1-inosine 5′-monophosphates (*N*1-IMPs). These small fragments inhibit hydrolysis of cADPR more efficiently than the parent cyclic compounds, with the best in the series demonstrating potent inhibition (IC_50_ = 7.6 µM). The lower molecular weight and relative simplicity of these compounds compared to cADPR make them attractive as a starting point for further inhibitor design.

## Introduction

**Figure 1:**
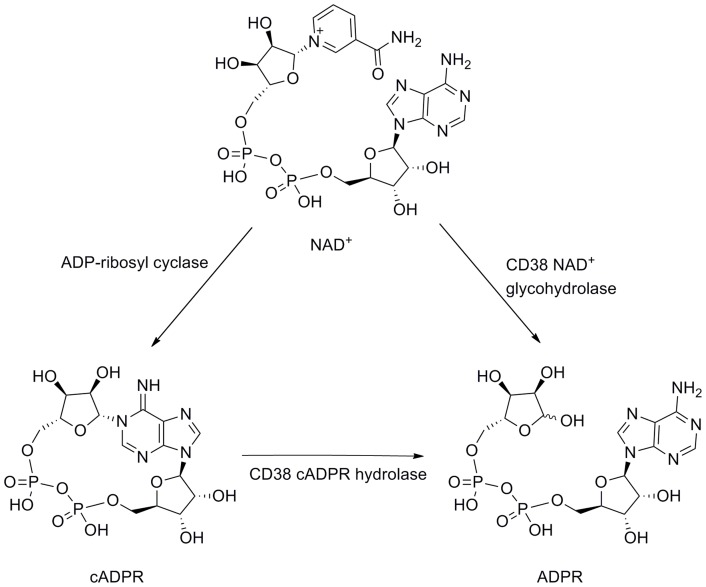
Conversion of NAD^+^ into cADPR and ADPR.

Cyclic ADP-ribose (cADPR, Figure 1) [1] is a second messenger that mobilizes intracellular Ca^2+^ in various types of cells including sea urchin eggs, T-cells and pancreatic β-cells. In biological systems, cADPR is synthesized enzymatically from nicotinamide adenine 5′-dinucleotide (NAD^+^) by the action of ADP-ribosyl cyclases. cADPR is readily hydrolyzed at the unstable *N*1 link to give ADP-ribose (ADPR) in both neutral aqueous solution and under physiological conditions [2]–[4], thus rendering chemical synthesis of non-hydrolyzable analogues attractive. In-depth reviews dealing with the chemistry of cADPR and the cADPR/Ca^2+^ signaling system have been published in recent years [5]–[11].

Human CD38 is a multifunctional protein that triggers proliferation and differentiation. The enzyme shares extensive sequence similarity with *Aplysia californica* ADP-ribosyl cyclase (ADPRC). It is principally an NAD^+^ glycohydrolase (NADase) that transforms NAD^+^ into ADPR (Figure 1), but CD38 is also able to produce a small amount of cADPR (ADP-ribosyl cyclase activity) and to hydrolyze cADPR into ADPR (cADPR hydrolase activity) [12], [13]. Studies have revealed the importance of CD38 in a range of diseases, *e.g.* CD38 is a marker of AIDS progression and a negative prognostic marker of chronic lymphocytic leukemia. Recently, CD38 has been shown to be critical for social behavior in mice [14]. Therefore, there is interest in developing new inhibitors of CD38 to provide structural clues for designing potential drug candidates for the treatment of CD38-related diseases. Thus far, only inhibitors of the NAD^+^ glycohydrolase activity of CD38 have been investigated. To date the best ones are mechanism-based covalent inhibitors, which bind to the active site of CD38. They have mainly been derived from NAD^+^, such as the nicotinamide ribose derivatives reported by Schramm *et al.* which exhibit K*_i_* values in the nanomolar range [15], [16]. Zhang *et al.* recently developed metabolically stable nicotinamide-based analogues which block endogenous CD38 activity in cells and tissues [17]. Lee *et al.* published a study on membrane permeable analogues, based on the nicotinamide motif, which are moderate (low mM) inhibitors of the enzymatic activities of CD38 and demonstrated their ability to relax agonist-induced muscle contraction [18]. Wall *et al.* reported a non-hydrolyzable NAD^+^ analogue as a competitive inhibitor of CD38, with an IC_50_ of about 100 µM [19]. Recently, other groups have successfully explored and reported non-nucleotide compounds as inhibitors of CD38. Kellenberger *et al.* showed that low micromolar concentrations of flavonoids inhibit CD38 [20]. Lately, Zhang and co-workers obtained a hit compound from commercially available libraries with an IC_50_ of 86 µM. Subsequent structural modification led to the most active non-covalent inhibitor of CD38 NADase activity thus far with an IC_50_ of 4.7 µM [21].

The crystallographic structure of the catalytic domain of CD38 as well as the mechanism of catalysis by which cADPR is metabolized have recently been elucidated using covalent inhibitors [22], [23]. Residue Glu-146 was identified as critical in regulating the multi-functionality of CD38-mediated NAD^+^ hydrolysis, ADP-ribosyl cyclase and cADPR hydrolysis activities [22], [24]. Glu-226 was identified as the catalytic residue as its mutation essentially eliminates catalytic activity [25]. cADPR forms two hydrogen bonds through *N*6 and *N*7 to Glu-146 and one hydrogen bond with Asp-155 through *N*6. Lately, Liu *et al.* have presented a comprehensive structural comparison study of CD38 and ADPRC [26]. Residue Phe-174 in ADPRC was identified as crucial in directing the folding of the linear substrate for cyclisation to occur. The equivalent residue Thr-221 in CD38 disfavors the folding process required for cyclization, resulting in the observed dominant NADase activity for this cyclase [26].

**Figure 2:**
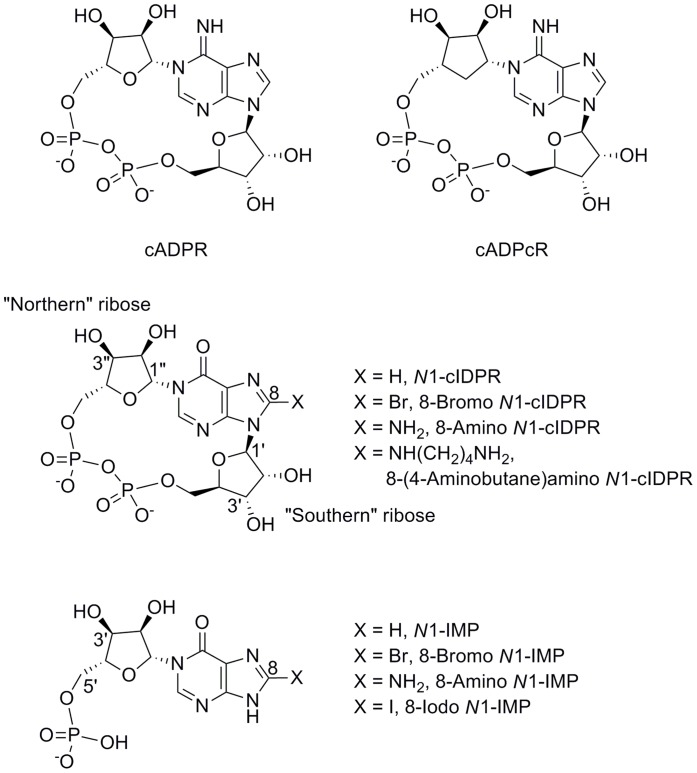
Structure and nomenclature of cADPR and analogues used in this study. The “northern” and “southern” riboses of the cyclic analogues are distinguished by adopting prime (′) and double prime (″) notation respectively for their sugar carbons.

Soaking of CD38 crystals with cADPR itself led to rapid hydrolysis of the ligand. Therefore, the crystal structure of cADPR was solved in complex with an inactive mutant of CD38 in which the catalytic residue Glu-226 had been mutated to Gln-226 (E226Q). In this catalytically inactive mutant, Gln-226 is not able to fulfill the usual role of Glu-226, in interacting with the “northern” ribose (for nomenclature of compounds see Figure 2). The crystal structure obtained with the E226Q mutant suggested that cADPR bound ‘less deeply’ in the active site, yet cADPR must be in close proximity to Glu-226 in the wild-type CD38 in order for catalysis to occur [27].

To explore the CD38:cADPR interaction, we previously designed a hydrolysis resistant cADPR analogue, cyclic inosine 5′-diphosphoribose (*N*1-cIDPR, Figure 2), [28], [29] that was co-crystallized with wild-type CD38. *N*1-cIDPR is a close structural analogue of cADPR in which an oxo group at position 6 replaces the amino group. It inhibits CD38-catalyzed cADPR hydrolysis with an IC_50_ of 276 µM. In Jurkat T-cells, Ca^2+^ release induced by *N*1-cIDPR is almost indistinguishable from Ca^2+^ release induced by cADPR [29]. A crystal structure of *N*1-cIDPR with wild-type CD38 [30] showed that *N*1-cIDPR binds in the active site, close to the catalytic residue Glu-226 and that the two hydroxyl groups of the “northern” ribose form two hydrogen bonds with this residue. In contrast to cADPR, the *O*6 atom of *N*1-cIDPR does not favor a hydrogen bond with Glu-146. This study has led to the most complete elucidation of the pathway of cADPR hydrolysis by CD38 yet and also facilitates, in principle, the possibility of structure-based design of novel inhibitors of CD38 using the *N*1-cIDPR template. We describe here the first successful steps on this route. We report X-ray crystal structures of two non-hydrolyzable inhibitors in complex with wild-type CD38, one using an 8-substituted *N*1-cIDPR analogue derived from structure-based design considerations and another using a different hydrolysis resistant ligand, cADP carbocyclic ribose (cADPcR, Figure 2). We also report the comparison of these crystal structures, which suggests the design of simple, novel fragments that could maintain key interactions with wild-type CD38. We describe the preparation of these compounds and their ability to inhibit cADPR hydrolysis.

## Experimental Procedures

### General

All reagents and solvents were of highest commercial quality and were used without further purification, unless described otherwise. H_2_O was of MilliQ quality. ^1^H, ^13^C, and ^31^P NMR spectra of final compounds were collected in D_2_O, either on a JEOL Delta machine at 270 MHz (^1^H) or 109 MHz (^31^P), or on a Varian Mercury system at 400 MHz (^1^H), 161 MHz (^31^P) or 100 MHz (^13^C). Chemical shifts (δ) are reported in parts per million (ppm). Abbreviations for splitting patterns are as follows: br, broad; s, singlet; d, doublet; t, triplet; m, multiplet. UV spectra were collected in aqueous solution on a Perkin Elmer Lambda EZ 201 or Lambda 3B spectrophotometer. HPLC analysis was carried out on a Waters 2695 Alliance module equipped with a Waters 2996 Photodiode Array Detector (210–350 nm). The chromatographic system consisted of a Hichrom Guard Column for HPLC and a Phenomenex Synergi 4u MAX-RP 80A column (150×4.60 mm), eluted at 1 mL/min with the following ion-pair buffer: 0.17% (*m/v*) cetrimide and 45% (*v/v*) phosphate buffer (pH 6.4) in MeOH. Preparative chromatography was performed on a Pharmacia Biotech Gradifrac system equipped with a peristaltic P-1 Pump and a fixed wavelength UV-1 Optical Unit (280 nm). Synthetic phosphates were assayed by an adaptation of the Briggs phosphate test [31].

### Molecular Modeling: cIDPR Analogues

Crystal structures of *N*1-cIDPR complexed with wild-type CD38 were obtained from the Protein Data Bank (PDB codes 2PGJ and 2PGL) [30]. Chain B was deleted from the crystal structure, as were all water molecules at a distance greater than 5Å from the ligand associated with chain A. Hydrogens were built onto the whole structure. All atoms with the exception of hydrogens were fixed in SYBYL aggregates, and hydrogens were energy minimized until convergence using the TRIPOS F.F force field with Gasteiger-Hückel charges applied. The docking calculations for *N*1-cIDPR and analogues were performed with the GOLD molecular docking package (version 3.0.1) [32]–[35]. The active site was defined as a 15Å sphere around the Thr-221 side chain oxygen atom and 50 attempts were computed for each inhibitor. From 50 docking attempts, one cluster was produced which mimicked the binding mode in the crystal structure. The ligand was energy minimized with the aromatic system in aggregates.

### *N*1-IMP Analogues

The 2PGJ and 3U4H crystal structures of human CD38 with cIDPR and 8-amino cIDPR, respectively, were passed through the Protein Preparation Wizard of the Schrödinger software running under Maestro version 9.3.026: the incorrect planar ribose in the 2PGJ structure was manually corrected so that the ribose had the appropriate structure and stereochemistry. Four cIDPR fragments, where the cIDPR “southern” ribose phosphate is deleted, were built in the Schrödinger software. The four compounds differed in the substituent at the 8-position: hydrogen, bromine, iodine or an amino group. The compounds were docked into the prepared structure using GOLD version 5.1. The binding site was defined as a sphere of 5Å radius centered on the centroid of the ligand in the prepared structure: the centroid of the docked ligand has to lie within this sphere. Each ligand was docked twenty-five times. Selected poses were merged with the protein into which they had been docked and the resulting complexes (with waters present) were put through 1,000 rounds of energy minimization using the Schrödinger software.

The ligand in the prepared 2PGJ and 3U4H crystal structures was modified so that the “southern” ribose phosphate was deleted. An amino group was added to the 8-position of the 2PGJ ligand. The resulting complexes were put through 1,000 rounds of minimization using the Schrödinger software. All figures were prepared using PyMOL.

In our experience the water molecules in the CD38 substrate binding site are very important when docking ligands. Without the water molecules present we find that GOLD is unable to reproduce the observed crystal structure poses and when docking ligands for which no crystal structure pose is available for comparison, docked poses obtained without water molecules present are highly variable and generally cannot explain the observed assay results.

### Chemistry

#### cIDPR analogues

8-amino *N*1-cIDPR [29], [36] and 8-bromo *N*1-cIDPR [28] were synthesized by a chemo-enzymatic procedure as previously described. cADPcR was prepared by total chemical synthesis as described [37]. Synthesis of 8-(4-aminobutane)amino *N*1-cIDPR was from 8-bromo *N*1-cIDPR using two methods as follows: *Method 1*: To a solution of diaminobutane (25 µL, 248 µmol) in MilliQ water (400 µL) was added 8-bromo *N*1-cIDPR (5 µmol). The reaction mixture was left standing for 10 days after which it was neutralized with 6N HCl. The reaction was diluted with MilliQ water to bring the conductivity down below 400 µS/cm and loaded onto an AG MP-1 ion exchange chromatography, eluting products with a linear gradient of 150 mM TFA at 3 mL/min. The appropriate fractions were collected and evaporated to dryness to afford the desired cyclic dinucleotide (2.59 µmol, 52%) as a glassy solid. ^1^H (400 MHz, D_2_O) δ 8.77 (s, 1H, H-2), 5.93 (br s, 1H, H-1″), 5.81 (d, *J*_1′,2′_ = 5.2 Hz, 1H, H-1′), 5.26 (app. t, *J*_2′,1′_ = *J*_2′,3′_ = 5.2 Hz, 1H, H-2′), 4.64 (app.t, *J* = 4.3 Hz, 1H, H-3′), 4.33–4.29 (m, 5H, H-5′a, H-5″a, H-2″, H-3″ and H-4″), 4.22–4.21 (m, 1H, H-4′), 4.07 (dd, *J*_5″b,5″a_ = 11.7 and *J*_5″b,4″_ = 3.9 Hz, 1H, H-5″b), 3.97–3.94 (m, 1H, H-5′b), 3.43–3.42 (m, 2H, CH_2_), 2.94–2.92 (m, 2H, CH_2_) and 1.68–1.67 (m, 4H, 2×CH_2_). ^31^P (109 MHz, D_2_O) δ –10.5 (br s) −11.2 (br s). HRMS (ES^+^) calcd for C_19_H_31_N_6_O_14_P_2_ 629.1368 (MH^+^) found 629.1357. UV (H_2_O) λ_max_ 265 nm (ε 13000). *Method 2*: A sealed flask containing a solution of diaminobutane (25 µL, 248 µmol) and 8-bromo *N*1-cIDPR (5 µmol) in MilliQ water (400 µL) was incubated at 70°C in a microwave for 1 h after which HPLC analysis showed total conversion of the starting material to a single product. The mixture was neutralized and product was purified as described in Method 1 to obtain the desired product in quantitative yield.

### *N*1-Inosine 5′-Monophosphate Analogues

#### 8-Bromo *N*1-IMP

8-Bromo *N*1-IMP was prepared as previously described [38].

#### 8-Iodo *N*1-IMP

A solution of 8-iodo *N*1-cIDPR [36] (5 µmol) in 0.2 M HCl (pH 1) was stirred at 60°C. After 24 hours, HPLC analysis showed completion of the reaction with the appearance of a new peak at 10.2 mins (R_t (8-I-cIDPR)_ = 11.3 mins). The reaction mixture was cooled to room temperature, neutralized by addition of 2 M NaOH and product purified on a reverse-phase column eluted with a gradient of MeCN in 0.05 M TEAB. The appropriate fractions were collected, evaporated and treated with chelex 100 to yield 8-iodo *N*1-IMP as a sodium salt (3.1 µmol, 62%) which showed ^1^H (400 MHz, D_2_O) δ 8.46 (s, 1H, H-2), 6.23 (d, 1H, *J*_1′,2′_ = 4.3 Hz, H-1′), 4.31 (app.t, 1H, *J*_2′,3′_ = *J*_2′,1′_ = 4.7 Hz, H-2′), 4.26 (app.t, 1H, *J*_3′,2′_ = *J*_3′,4′_ = 5.1 Hz, H-3′), 4.26–4.25 (m, 1H, H-4′), 4.09–4.04 (m, 1H, H-5′a) and 4.00–3.95 (m, 1H, H-5′b). ^13^C (100 MHz, D_2_O) δ 159.2 (C-6), 156.4 (C-4), 142.1 (C-2), 125.7 (C-5), 110.3 (C-8), 88.1 (C-1′), 83.7 (C-4′, *J* = 8.4 Hz), 75.1 (C-2′), 69.5 (C-3′) and 62.9 (C-5′). ^31^P (161 MHz, D_2_O) δ 3.8. HRMS (ES^−^) calculated for C_10_H_11_IN_4_O_8_P 472.9365 (M−H)^−^ found 472.9370. UV (H_2_O) λ_max_ 261 nm (ε 16500).

#### *N*1-IMP

To a solution of 8-bromo *N*1-IMP (3 µmol) and NaHCO_3_ (30 mg) in water/EtOH (1∶0.5 mL) was added Pd/C (2 mgs). The mixture was stirred under a hydrogen atmosphere for 5 h after which HPLC analysis showed a new peak at 8.3 mins. The palladium was removed by filtration and the filtrate was evaporated leaving a residue which was purified by reverse-phase column eluted with a gradient of MeCN in 0.05 M TEAB to yield the desired product as a glassy solid (2.1 µmol, 70%). ^1^H (270 MHz, D_2_O) δ 8.57 (s, 1H, H-2), 7.86 (s, 1H, H-8), 6.32 (d, 1H, *J*_1′,2′_ = 4.2 Hz, H-1′), 4.45 (dd, 1H, *J*_2′,3′_ = 5.0 and *J*_2′,1′_ = 4.2 Hz, H-2′), 4.42 (app.t, 1H, *J*_3′,2′_ = *J*_3′,4′_ = 5.0 Hz, H-3′), 4.30–4.27 (m, 1H, H-4′) and 4.09–3.97 (m, 2H, H-5′). ^13^C (100 MHz, D_2_O) δ 154.9 (C-6), 151.1 (C-2), 142.2 (C-8), 88.3 (C-1′), 83.7 (d, *J* = 8.8 Hz, C-4′), 75.2 (C-2′), 69.7 (C-3′) and 63.0 (C-5′). ^31^P (161 MHz, D_2_O) δ 0.9. HRMS (ES^−^) calcd for C_10_H_12_N_4_O_8_P 347.0398 [(M−H)^−^] found 347.0396. UV (H_2_O) λ_max_ 262 nm (ε 12500).

#### 8-Amino *N*1-IMP

A solution of 8-azido *N*1-cIDPR [29] (5 µmol) in 0.2 M HCl (pH 1, made up in D_2_O) was stirred at 60°C. After 24 hours, ^31^P NMR showed disappearance of the pyrophosphate signal around δ -10 ppm and formation of two peaks between 0 and 1 ppm, characteristic of monophosphates. The reaction mixture was cooled to room temperature, neutralized by addition of 0.1 M NaOH and product purified on a C18 semi-preparative column eluted with a gradient of MeCN in 0.1 M TEAB. The appropriate fractions were collected, evaporated and the residue was dissolved in 0.05 M TEAB to which was added dithiothreitol (10 mgs). The reaction mixture was stirred at room temperature in the dark for 3 h, after which HPLC analysis showed the reaction was complete. The solvent was removed and the residue applied to a C18 semi-preparative column eluted with a gradient of MeCN in 0.1 M TEAB to afford the desired monophosphate (1.1 µmol, 22% over 2 steps). ^1^H (500 MHz, D_2_O) δ 8.49 (s, 1H, H-2), 6.32 (brs, 1H, H-1′), 4.33–4.36 (m, 2H, H-2′ and H-3′), 4.25 (brs, 1H, H-4′) and 4.05–4.14 (m, 2H, H-5′). ^13^C (125 MHz, D_2_O) δ 161.4 (C-6), 155.7 (C-4), 152.8 (C-5), 143.6 (C-2), 112.3 (C-8), 89.0 (C-1′), 83.1 (d, *J* = 8.9 Hz, C-4′), 75.0 (C-2′), 69.0 (C-3′) and 63.4 (d, *J* = 4.5 Hz, C-5′). ^31^P (202 MHz, D_2_O) δ 0.3. HRMS (ES^−^) calcd for C_10_H_13_N_5_O_8_P 362.0507 (M−H)^−^ found 362.0509. UV (H_2_O) λ_max_ 265 nm (ε 11000).

#### Protein production, crystallization, complex formation and data collection

Expression, purification, and crystallization of wild-type CD38 protein were performed using procedures as previously described [22]. The CD38/8-amino *N*1-cIDPR complex was obtained by incubating pre-formed wild-type CD38 crystals with 30 mM 8-amino *N*1-cIDPR for 1–2 min at 4°C in soaking solution (10% PEG 4000, 100 mM MES, pH 6.0, and 30% glycerol). The CD38/cADPcR complex was obtained by incubating pre-formed CD38 crystals in 33 mM cADPcR for 100 min at 4°C in soaking solution (10% PEG 4000, 100 mM MES, pH 6.0, and 30% glycerol). X-ray diffraction data were collected at the Cornell High-Energy Synchrotron Source (CHESS) A1 station under the protection of a liquid nitrogen cryo-stream at 100 K. Diffraction images were integrated, scaled, and merged by using the program HKL2000 [39]. Data reduction statistics are listed in Table S1.

### Structure Refinement

Structures were refined by REFMAC [40] and PHENIX [41] with the starting model derived from the CD38/*N*1-cIDPR complex (PDB code: 2PGJ) [30]. There are two CD38 molecules in the crystallographic asymmetric unit. The 8-amino *N*1-cIDPR and cADPcR molecules were built in O [42] based on the structure of *N*1-cIDPR and the σA weighted Fo-Fc difference electron densities. Solvents were added automatically by ARP/wARP [43] and manually inspected/modified under the program O. At the end of refinements, TLS refinement implemented in PHENIX was introduced to model the data anisotropy. Refinement statistics are also listed in Table S1.

### Structural Data

The atomic coordinates and structure factors of CD38/8-amino *N*1-cIDPR and CD38/cADPcR have been deposited with the Protein Data Bank (www.pdb.org) with the accession codes 3U4H and 3U4I, respectively.

### Enzymatic Assay for cADPR Hydrolysis

The inhibition of cADPR hydrolysis by various concentrations of inhibitor (0–1 mM) was determined by incubating 1 µM cADPR with 2 µg/mL of CD38 for 10 min at 20–24°C in 25 mM sodium acetate, pH 4.5. The reaction was stopped by the addition of 150 mM HCl. The precipitated protein was filtered, and the pH was neutralized with Tris base. After diluting the mixture 20-fold, the concentration of the unhydrolyzed cADPR present in the diluted reaction mixture was assayed by the fluorimetric cycling assay as previously described [44].

### Enzymatic Assay for NAD^+^ Hydrolase Activity

The inhibition of CD38 NAD^+^ glycohydrolase activity by 8-NH_2_-cIDPR (0–1 mM) was determined by incubating 1 µM NAD^+^ with 0.1 µg/mL CD38 for 1 min at 20–24°C in 25 mM sodium acetate pH 4.5. The reaction was stopped by addition of 150 mM HCl, filtered and assayed for NAD^+^ by the fluorimetric cycling assay as previously described [44].

## Results and Discussion

### Molecular Modeling to Predict Structural Modifications

**Figure 3:**
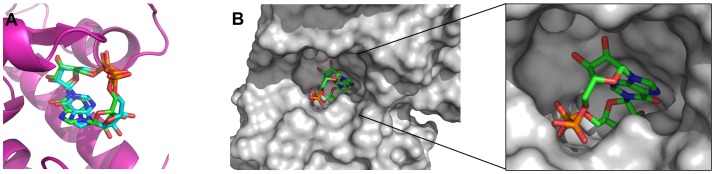
*N*1-cIDPR in the CD38 binding site. (A) Comparison between the docked model of *N*1-cIDPR (carbons in cyan) and the crystal binding model (carbons in green). The color scheme is O, red; N, dark blue and P, orange and (B) surface view of the active site pocket of the crystal structure of CD38 with *N*1-cIDPR bound inside.

We previously reported the crystal structure of *N*1-cIDPR with wild-type CD38 (PDB code 2PGL) [30]. To compare the binding mode of *N*1-cIDPR and its analogues with CD38, they were docked in a similar manner into 2PGL with *N*1-cIDPR removed using the flexible docking program GOLD [32]–[35]. When *N*1-cIDPR is redocked back into the vacant active site with the structural water molecules present, its pose is almost identical to the position of the ligand in the original protein crystal structure, providing confidence that docking could be predictive for other ligands using this model (Figure 3A). The surface view of the active site complexed with the ligand reveals a small pocket around the 8-position of the purine ring and a second much larger pocket of vacant space around the “southern” ribose which could be exploited to design potential inhibitors of CD38 (Figure 3B).

**Figure 4:**
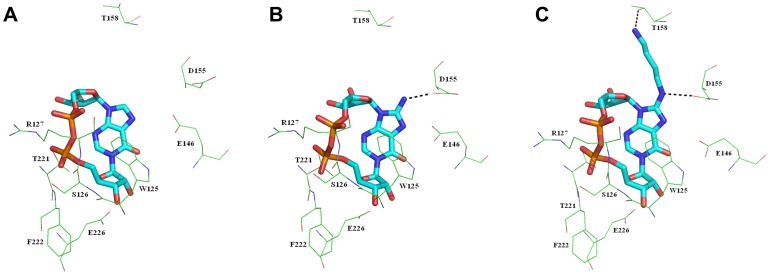
Highest scoring docked conformation of *N*1-cIDPR (A), 8-amino *N*1-cIDPR (B) and 8-(4-aminobutane)amino *N*1-cIDPR (C). The ligands are shown as sticks and the residues as lines. Color codes of the atoms of the ligand are C, cyan, O, red; N, dark blue and P, orange. Dashed black lines show the hydrogen bond interactions between the ligands and the enzyme. Hydrogen atoms are not shown for clarity.

The stability of *N*1-cIDPR towards chemical hydrolysis was recently exploited to generate a series of analogues from the synthetically versatile parent compound 8-bromo *N*1-cIDPR [29]. Docking was used to determine the likely binding mode and ligand contacts arising from substitution of the bromine of 8-bromo *N*1-cIDPR by -NH_2_ at C-8 of the purine ring. The main interactions predicted for the cyclic backbone are with amino acids Trp-125, Ser-126, Arg-127, Thr-221 and Phe-222 (binding motif) as well as Glu-146 (regulatory motif) and Glu-226 (catalytic residue). These interactions are also observed in the 2PGL crystal structure with *N*1-cIDPR co-crystallized. The importance of many of these residues is highlighted by the fact that they are evolutionarily conserved – see Figure S4 for an alignment of CD38 from seven species. The distance between the hydrogen of the amino group of 8-amino *N*1-cIDPR and the carboxylate oxygen of Asp-155 is 2.63 Å, which suggests that hydrogen bonding in this area is possible (Figure 4A and B). It was thus envisaged that the presence of an amino group might increase affinity of 8-amino *N*1-cIDPR for the active site relative to *N*1-cIDPR with resulting enhanced inhibitory activity towards CD38. A second ligand, 8-(4-aminobutane) amino *N*1-cIDPR, was designed to explore the possibility of a yet stronger ionic interaction with Asp-155. It was envisaged that a diaminobutane group at this position might afford a stronger overall interaction between the ligand and protein, as the primary amine at the chain terminus could interact through hydrogen bonding to Thr-158 in addition to a hydrogen bond between the C-8 amino group and Asp-155, as already predicted by the 8-amino *N*1-cIDPR/CD38 docking. The -NH(CH_2_)_4_NH_2_ substituent could be incorporated during binding in two modes, depending on the orientation of the -NH hydrogen. The model was therefore built in the conformation likely to form the most hydrogen bonds with CD38. The docked distance between the hydrogen of the secondary amino group and the carboxylate oxygen of Asp-155 was 2.40 Å; while the NH_2_-CO_2_- distance between the primary amino group and Thr-158 was 2.11 Å, indicating that two hydrogen bonds can in principle be derived from the C-8 (aminobutyl)amino side chain (Figure 4C).

### Synthesis and Characterization of 8-(4-aminobutane)Amino *N*1-cIDPR

**Figure 5:**
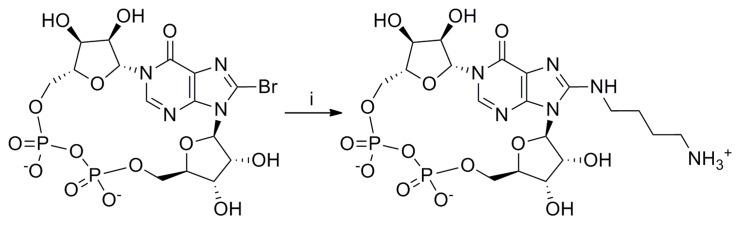
Preparation of 8-(4-aminobutane)amino *N*1-cIDPR. Reagents and conditions: i) diaminobutane, MilliQ, rt, 10 days or diaminobutane, MilliQ, microwave, 70°C, 1 h.

Few cADPR analogues bearing an extended amino chain at C-8 have been synthesized, presumably due to the instability of cADPR itself towards hydrolysis. The first, 8-(6-aminohexyl)amino cADPR, was prepared by direct incubation with *Aplysia* cyclase of the commercially available 8-(6-aminohexyl)amino NAD^+^ [5]. In contrast, our route depends upon the excellent stability of the *N*1-cIDPR template, on which we have previously demonstrated efficient displacement of the 8-bromo atom [29]. A large excess of diaminobutane is required to displace the bromine and drive the reaction to completion over a 10-day period (Figure 5), characterized by a shift in UV absorption from 255 to 265 nm. The identity of the expected 8-(4-aminobutane)amino *N*1-cIDPR was confirmed by spectroscopy. Mass spectroscopy gives a peak with *m/z* value of 629.1 (MH)^+^ consistent with the expected product. The ^1^H NMR spectrum is also in agreement with the proposed cyclic structure with one broad singlet at 5.93 and a doublet at 5.81 for anomeric protons H-1″ and H-1′ respectively. In addition, multiplets at 3.4, 2.1 and 1.6 ppm indicate the presence of the alkyl chain. Using microwave technology the yield of the displacement reaction could be improved from 52% to quantitative. Moreover, the reaction could be carried out in 1 h as opposed to 10 days using the unassisted route. In addition to its application as a CD38 inhibitor in this study, we anticipate that this compound should provide an ideal starting point from which an affinity chromatography column for isolation of cADPR-binding proteins could be derived.

### Synthesis of *N*1-inosine 5′-monophosphate Analogues

**Figure 6:**
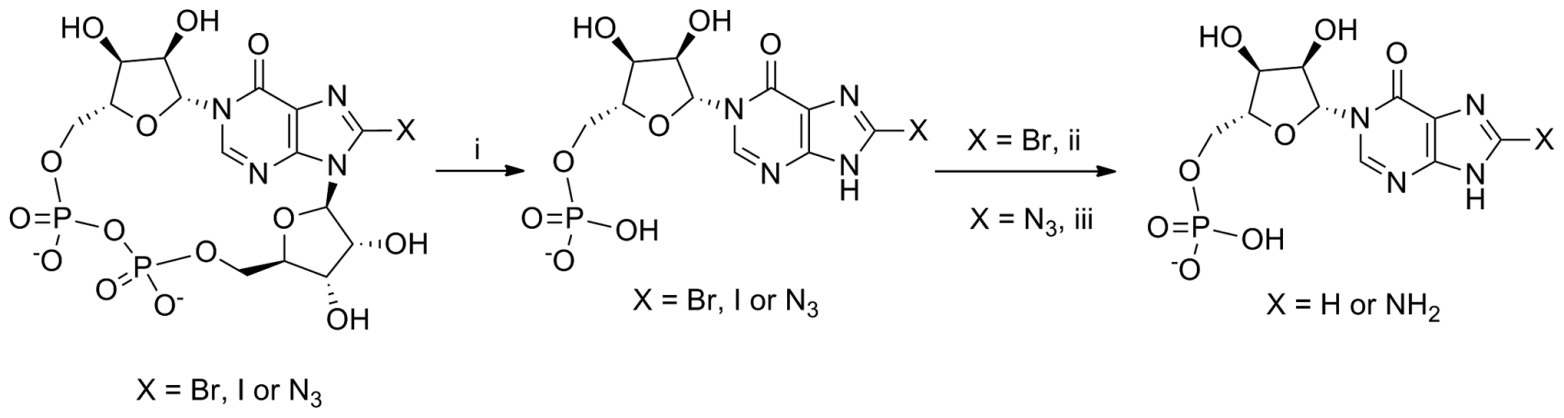
Preparation of *N*1-IMP compounds. Reagents and conditions: i) 0.2 M HCl, 60°C, 24 h; ii) Pd/C, H2, NaHCO3, MilliQ-EtOH, 5 h; iii) dithiothreitol, 0.05 M TEAB, 3 h.

While studying the stability of 8-bromo *N*1-cIDPR it was found that, on prolonged heating at acidic pH, it degraded very cleanly to generate a novel class of nucleotide where the *N*9 glycosidic bond in the cyclic compound was cleaved in preference to the *N*1 bond; leaving 8-bromo *N*1-IMP (Figure 6) [38]. Similar treatment of 8-iodo *N*1-cIDPR afforded 8-iodo *N*1-IMP. Subsequent palladium catalyzed hydrogenation of 8-bromo *N*1-IMP afforded the parent compound *N*1-IMP. However, 8-amino *N*1-cIDPR could not be degraded in the same manner as 8-bromo *N*1-cIDPR, presumably due to the free amino group. It required heating for a longer period at acidic pH which, over time, ended up degrading the product fully to a complex mixture of compounds. An alternative method to obtain the fragment is to degrade the parent 8-bromo *N*1-cIDPR followed by treatment with NaN_3_ to produce the 8-azido IMP which in turn could be reduced to the amino compound. To our surprise, the nucleophilic displacement step by NaN_3_ proved unsuccessful. Various changes in the reaction conditions; converting the starting material from the triethylammonium salt to the free acid, or varying the reaction temperature (80 to 120°C) were fruitless. When treating both 8-bromo *N*1-cIDPR and 8-bromo *N*1-IMP in parallel under identical conditions, only 8-bromo *N*1-cIDPR was converted to the desired azido product, therefore ruling out potential chemical contamination. Given that the only difference between the two starting materials is the *N*9 position (which is not alkylated in 8-bromo *N*1-IMP), we speculated that in this particular case the C-8 position is not electrophilic enough to allow the azide to attack and displace the bromine atom. Instead, the precursor 8-azido *N*1-cIDPR [29] was degraded under acidic conditions to prepare 8-azido *N*1-IMP which, upon treatment with dithiothreitol, gave the desired 8-amino *N*1-IMP. Although we realize this is not an elegant or cost-effective way of preparing these fragments, a direct synthetic approach is underway and will be reported in due course.

### Structure of 8-amino *N*1-cIDPR Complexed with Wild-type CD38

**Figure 7:**
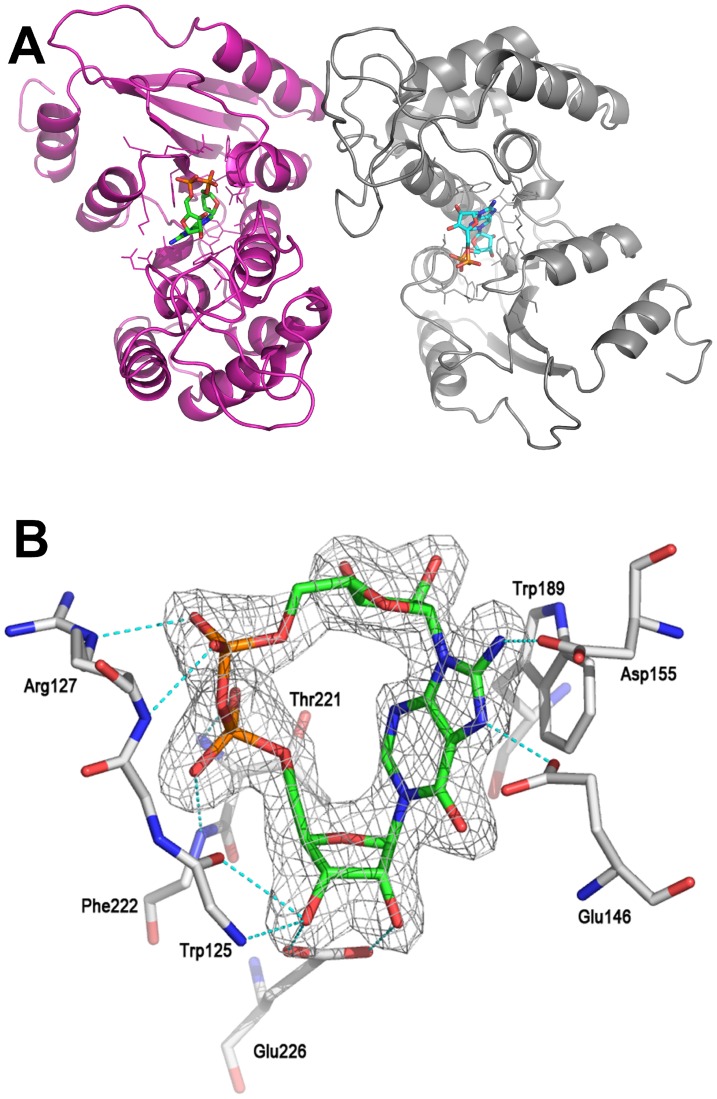
The crystal structure of CD38 with 8-amino *N*1-cIDPR. (A) View of the overall structure of the CD38/8-amino *N*1-cIDPR complex showing a ligand present in each molecule of CD38. (B) Structure of 8-amino *N*1-cIDPR complexed with wild-type CD38. The σA weighted different electron density is shown as gray isomesh contoured at 2.5σ. H-bonds are shown as dashed lines and colored in cyan.

Preformed crystals of wild-type CD38 were soaked with a solution of 8-amino *N*1-cIDPR. Crystals usable for data collection were obtained and examined by X-ray crystallography to determine the structure of the enzymatic domain of the CD38/ligand complex. The structure was refined to a resolution of 1.87Å and contained two molecules of CD38 in the asymmetric unit with 8-amino *N*1-cIDPR present in the respective active sites (Figure 7A). The 2″- and 3″-OH groups on the “northern” ribose form two hydrogen bonds with the carboxylate group of Glu-226. In addition to Glu-226, the pyrophosphate group interacts with a number of previously identified amino acids including Trp-125, Arg-127, Thr-221 and Phe-222 [27]. Importantly, the 8-amino group forms a hydrogen bond with the carboxylate group of Asp-155 as predicted by our modeling studies (Figure 7B).

### Structure of cADPcR Complexed with Wild-type CD38

**Figure 8:**
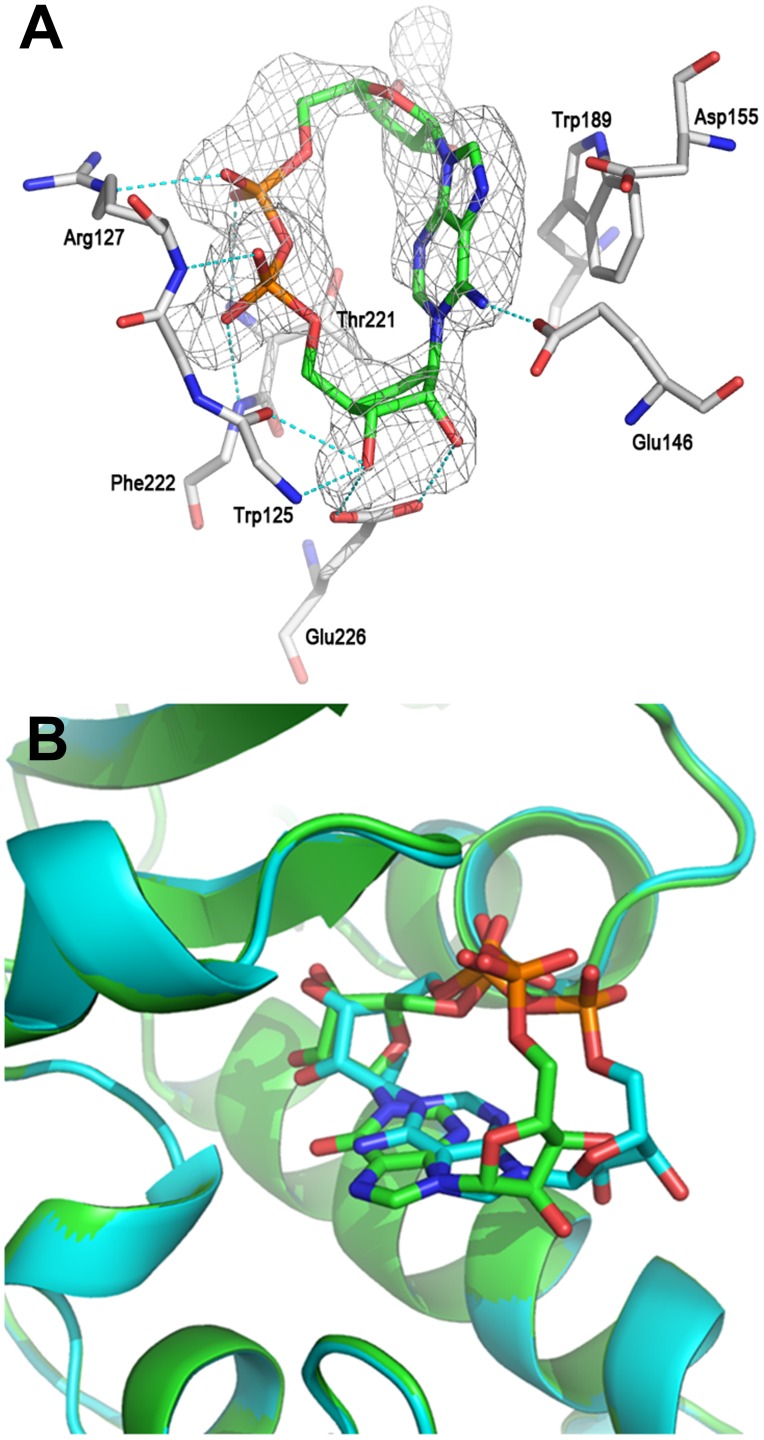
The crystal structure of CD38 with cADPcR. (A) Structure of cADPcR complexed with wild-type CD38. The σA weighted Fo-Fc different electron density is shown as gray isomesh contoured at 2.5σ. H bonds are shown as dashed lines and colored in cyan (B) Binding comparison between *N*1-cIDPR (carbons in green) and cADPcR (carbons in cyan) complexed with CD38, showing overlap of their “northern” ribosyl phosphate motifs.

Cyclic ADP-carbocyclic ribose was synthesized as a stable mimic of cADPR in which the oxygen of the “northern” ribose is replaced by a methylene group [37]. This small change, by removing the ability to form an oxocarbenium ion in the “northern” ribose, confers stability towards chemical and biochemical hydrolysis in a different fashion to that afforded by *N*1-cIDPR, and cADPcR has also proved to be a potent Ca^2+^ release agent in sea urchin egg homogenate. Moreover, unlike cIDPR, cADPcR retains the *N*6-amino group of cADPR. As with 8-amino *N*1-cIDPR, preformed crystals of CD38 were soaked in a solution of cADPcR at pH 6 to form the complex. The structure of the CD38/cADPcR complex obtained was refined to 2.1 Å resolution. As already seen for *N*1-cIDPR or *N*7-cGDPR [27], [30], cADPcR can be detected in only one of the two crystallographic asymmetric units. Like *N*1-cIDPR, cADPcR can reach the catalytic residue Glu-226 and form two hydrogen bonds with it through the two hydroxyl groups of the carbocyclic ribose. The diphosphate group is stabilized through a number of H-bonds with residues Trp-125, Arg-127, Thr-221 and Phe-222 (Figure 8A). The adenine ring of cADPcR interacts with Glu-146 through a hydrogen bond with the *N*6 nitrogen and with Trp-189 through hydrophobic interaction. As expected, Asp-155 does not appear to be directly involved in the stabilization of the cADPcR/CD38 complex.

*N*1-cIDPR was thus the first analogue that could reveal the likely cADPR binding mode in the wild-type enzyme, but misses the *N*6-amino group. It is pleasing now to see with cADPcR that an analogue that preserves this key cADPR motif still binds essentially in the same location.

### Attempt to Obtain Structural Complexes of 8-bromo *N*1-cIDPR and 8-(4-aminobutane)Amino *N*1-cIDPR with Wild-type CD38

The co-crystal structure of 8-amino *N*1-cIDPR bound to wild-type CD38 encouraged us to attempt crystallization with other analogues which had been modified in the 8-position. However, soaking 8-(4-aminobutane) amino *N*1-cIDPR ligand with CD38 crystals resulted in no electron density in the active site, despite our prediction that the protonated amine may pair with Asp-155 and Thr-158. We suggest that this motif may also generally disturb the architecture of the protein too much as the ligand seeks out other potential ionic charged partners. A similar problem was encountered in attempts to isolate a productive complex with 8-bromo *N*1-cIDPR, suggesting either weaker binding for this ligand, or even possibly, cleavage by the protein. However, the latter seems unlikely, as incubation of 8-bromo *N*1-cIDPR with CD38 revealed no change in the nucleotide profile when analyzed by HPLC after 18 h (data not shown). Similar stability was observed by Kirchberger *et al.* when studying the metabolic stability of *N*1-cIDPR derivatives [45]. Despite this, we note *vide infra* that 8-bromo *N*1-cIDPR does bind with an IC_50_ actually better than *N*1-cIDPR, but that the diaminobutane derivative binds very poorly.

### Inhibition of Wild-type CD38-catalyzed cADPR Hydrolysis by *N*1-cIDPR Analogues

**Figure 9:**
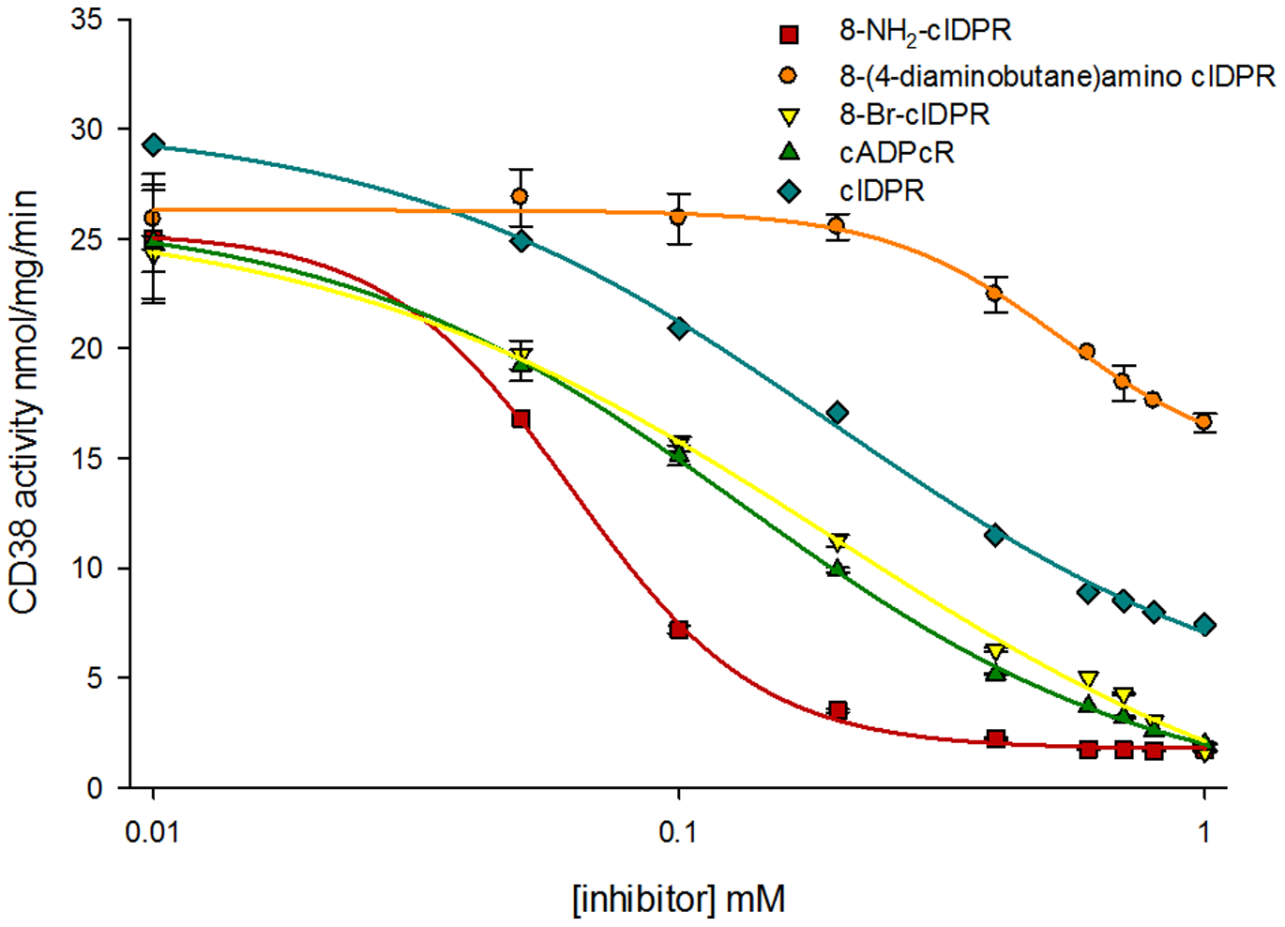
Inhibition of cADPR hydrolysis by synthetic inhibitors. CD38 hydrolysis activity plotted against analogue concentration.

*N*1-cIDPR binds in the active site of wild-type CD38, therefore blocking entry and subsequent hydrolysis of cADPR, with an IC_50_ of 276 µM. Our modeling studies, as well as the X-ray data obtained for 8-amino *N*1-cIDPR and cADPcR, suggest that the active site of CD38 has good affinity for both classes of non-hydrolyzable cADPR analogue; and that they should block entry of cADPR into the catalytic pocket. The inhibition of CD38-catalyzed cADPR hydrolysis was thus measured for all cyclic analogues explored in this study (Figure 9).

**Figure 10:**
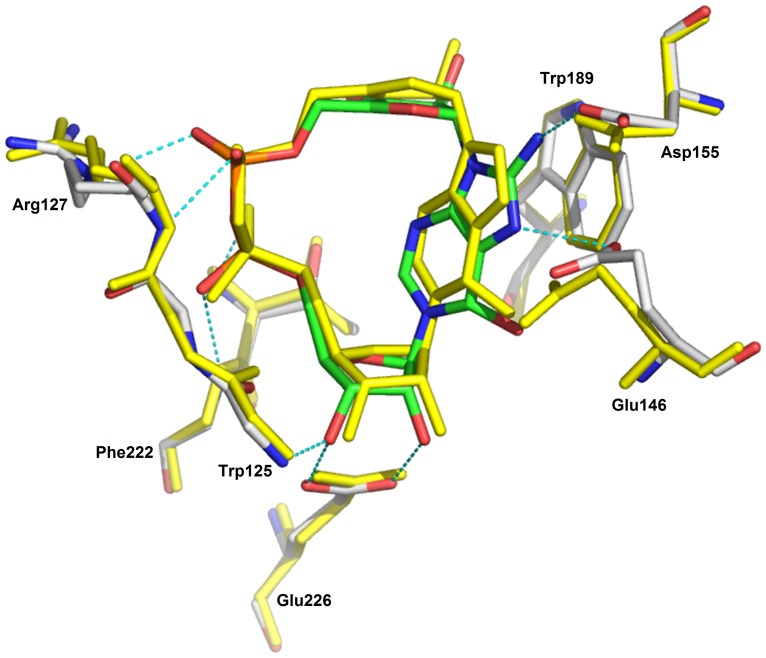
Superimposition of the CD38/*N*1-cIDPR complex (shown in yellow sticks) with the CD38/8-amino *N*1-cIDPR.

The higher definition of the X-ray maps for the 8-amino *N*1-cIDPR/CD38 complex, in addition to the extra hydrogen bond between the amino group and the carboxylate of Asp-155, indicate a likely increase in affinity of this ligand for the protein over the parent *N*1-cIDPR. Indeed, 8-amino *N*1-cIDPR inhibited cADPR hydrolysis with an IC_50_ of 56 µM, over 5 times better than that obtained for *N*1-cIDPR (Table 1). The crystal structures of *N*1-cIDPR and 8-amino *N*1-cIDPR in complex with CD38 were aligned based on their active site residues. Both structures are very similar except for the position of Glu-146. In the 8-amino *N*1-cIDPR complex, the side chain of Glu-146 is displaced significantly to form an H-bond with the *N*7 atom of the hypoxanthine ring (Figure 10; such movement of Glu-146 could not have been readily predicted from the non-dynamic docking protocol used in our modeling studies). The conformational change of Glu-146 probably reflects the enhanced affinity between the inhibitor and CD38. The high resolution crystal structure of cADPcR bound in the active site illustrates two hydrogen bonds with Glu-226 (Figure 8). Furthermore, the presence of the *N*6 atom (as in cADPR) favors hydrogen bonding to Glu-146, which may explain the improved affinity of cADPcR for CD38, compared to *N*1-cIDPR, resulting in improved inhibition of cADPR hydrolysis (IC_50_ = 129 µM vs 276 µM).

**Table 1:** Inhibition of cADPR hydrolysis by CD38 for cIDPR and *N*1-IMP analogue.

*Compound*	IC_50_
**cIDPR analogues**	
8-Amino *N*1-cIDPR	56 µM ±9 µM
cADPcR	129 µM ±12 µM
8-Bromo *N*1-cIDPR	158 µM ±13 µM
*N*1-cIDPR	276 µM ±10 µM [30]
8-(4-Aminobutane)	>1 mM
amino *N*1-cIDPR	
***N*** **1-IMP analogues**	
8-Iodo *N*1-IMP	>1 mM
8-Bromo *N*1-IMP	201 µM ±41 µM
*N*1-IMP	14 µM ±5 µM
8-Amino *N*1-IMP	7.7 µM ±0.2 µM

8-(4-Aminobutane)amino *N*1-cIDPR is the poorest inhibitor of cADPR hydrolysis with an IC_50_ of approximately 2 mM (estimated by projection). As shown in Figure 3B, there is only a small pocket around the 8 position of the purine ring and it might be that this space is too tight to accommodate the diaminobutane chain. Indeed, as shown in Figure 4C, the diaminobutane group appears to protrude from the active site.

Our docking studies indicate that 8-bromo *N*1-cIDPR should fit in the active site of CD38 in a similar manner to 8-amino *N*1-cIDPR (data not shown). Since the bromo and amino groups are of similar size, it may be that they both sit well in the pocket around the C-8 position and therefore provide a better fit in the active site than *N*1-cIDPR itself. This may explain the improved IC_50_ of 8-bromo *N*1-cIDPR towards cADPR hydrolysis by CD38 (158 µM) relative to *N*1-cIDPR.

**Figure 11:**
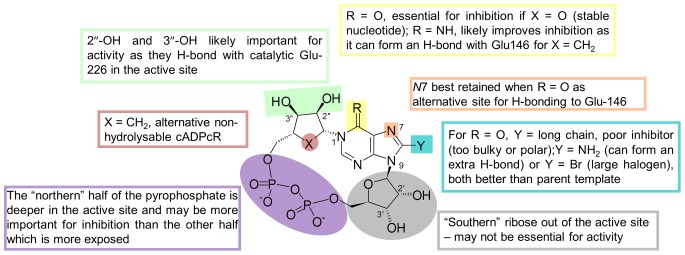
Preliminary structure-activity relationship for inhibitors of cADPR hydrolysis by CD38.

Based on these results, a very preliminary SAR can be constructed (Figure 11). The oxo group at the 6-position of the purine ring appears to be useful for inhibitory activity, primarily due to conferring resistance to CD38-catalyzed hydrolysis. When the oxo group is replaced by an imino group (as in cADPR, the parent compound), inhibition is improved providing the nucleotide is non-hydrolyzable as in cADPcR where the “northern” ribose is replaced by a carbocycle. This is perhaps unsurprising since the imino group can form a hydrogen bond with Glu-146.

There is a small pocket around the 8-position of the purine ring and groups at this position appear to be crucial for improved inhibitory activity. An amino group, as predicted by modeling and confirmed by crystallography, seems ideal as it is small enough to fit in the pocket and forms a new hydrogen bond with Asp-155.

### Inhibition of NADase Activity of Wild-type CD38

The majority of published studies regarding CD38 inhibition have focused on the ability of compounds to inhibit the NADase activity of CD38, rather than its cADPR hydrolase activity. In order to compare our inhibitors, we therefore tested 8-NH_2_-*N*1-cIDPR (our best cyclic inhibitor with a cADPR hydrolase IC_50_ of 56 µM) as an inhibitor of NADase activity, and found it to have an IC_50_ of 21 µM (Figure S1). This places it among the more potent inhibitors in this class. Furthermore, it suggests that the compounds in this study may have wider application as general CD38 inhibitors and that, in this case, they do not demonstrate specificity for the cADPR hydrolase activity of CD38, despite their similarity in structure to cADPR.

### *N*1-Inosine 5′-monophosphate Fragments as Inhibitors of cADPR Hydrolysis

**Figure 12:**
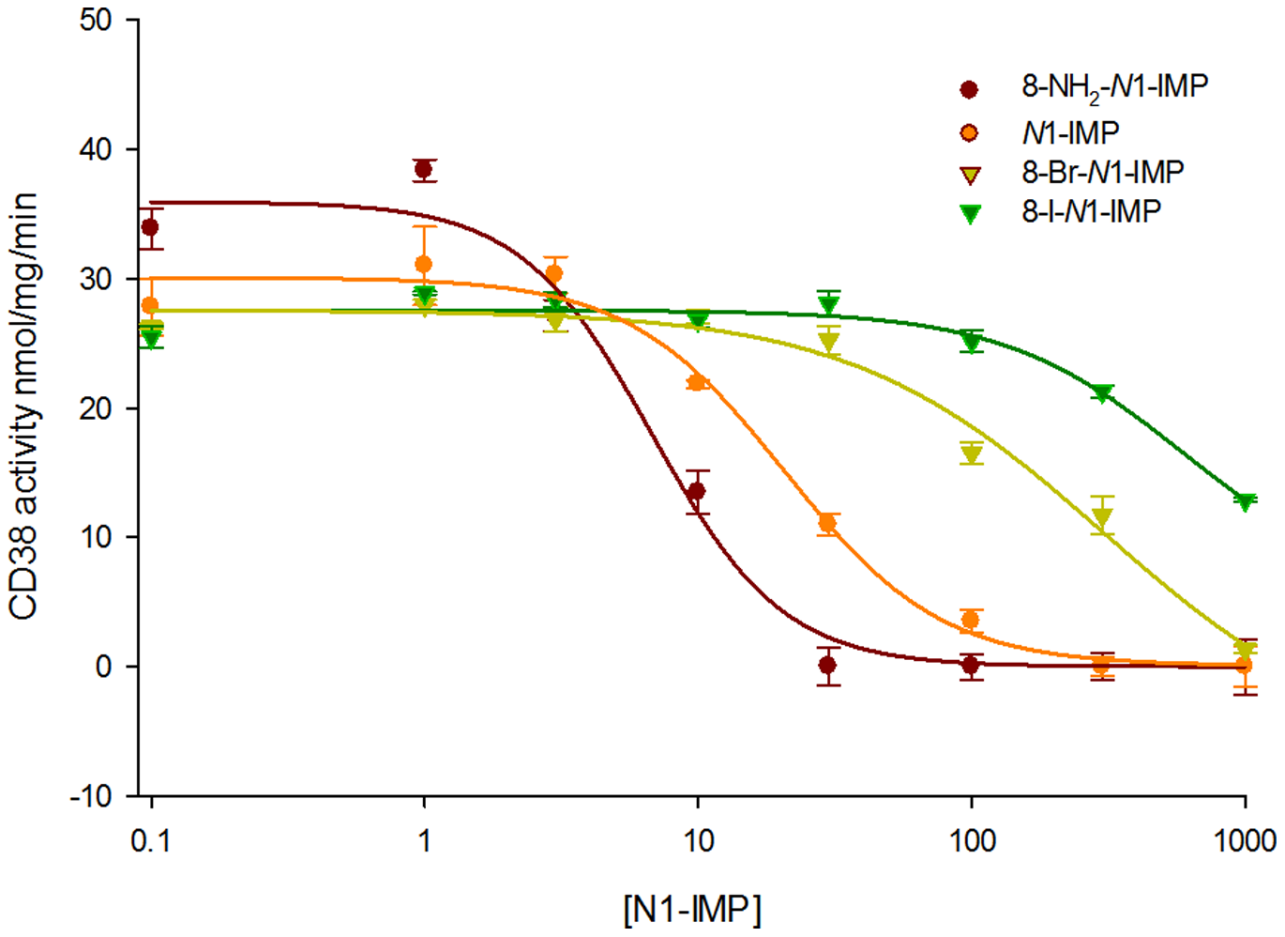
Inhibition of cADPR hydrolysis by *N*1-IMP fragments.

A binding comparison between *N*1-cIDPR and cADPcR complexed with wild-type CD38 shows that both ligands bind to the active site in a similar position and orientation. Indeed, the “northern” ribose monophosphate of *N*1-cIDPR and the carbocyclic ribose monophosphate of cADPcR overlap whilst the rest of the molecule is accommodated in a potentially more flexible fashion across the open face of the pocket (Figure 8B). This suggests that the “northern” ribose part of the cyclic dinucleotide (ribose and/or carbocycle) is more important in binding than the “southern” part. To further investigate this hypothesis, we tested our small series of *N*1-inosine 5′-monophosphate (*N*1-IMP or, more strictly, *N*1-hypoxanthine ribose 5′-monophosphate - note that these compounds correspond formally to the *N*1-inosine 5′′-monophosphate fragment when using the cyclic nucleotide notation adopted herein) fragments as inhibitors of CD38-catalyzed cADPR hydrolysis (Figure 12). 8-Bromo *N*1-IMP showed an IC_50_ of 201 µM which is consistent with what was observed for the cyclic compound (Table 1). In contrast, 8-iodo *N*1-IMP had a much higher IC_50_ (estimated around 1.24 mM by projection). This may suggest that, similar to the 8-diaminobutane chain, the 8-iodo substituent is too large to fit comfortably into the binding site pocket. Surprisingly, the parent *N*1-IMP was nearly 20 fold better than its cyclic counterpart with an IC_50_ of 14 µM (*cf. N*1-cIDPR 276 µM). This was unexpected; for the series of cyclic compounds an opposite trend was observed in which exploiting the binding pocket (for example with the 8-bromo substituent) led to better inhibition of cADPR hydrolysis. The 8-amino *N*1-IMP was also an order of magnitude better than its cyclic counterpart (7.6 µM *cf.* 8-NH_2_-*N*1-cIDPR at 56 µM), making this the most potent inhibitor of cADPR hydrolase activity developed in this study. In this case, we suspect that this may be attributed to the re-introduction of the hydrogen bond to Asp-155.

To further investigate the binding of the *N*1-IMP fragments, we docked the four ligands into two crystal structures of CD38; the 2PGJ structure with *N*1-cIDPR as ligand, and the 3U4H structure reported here with 8-NH_2_-cIDPR as the ligand. When docked into the 2PGJ structure, all four *N*1-IMP ligands resulted in almost identical docked poses (exemplified by 8-amino *N*1-IMP, Figure S2). This docking suggests that the *N*1-IMP ligands do not penetrate as far into the binding site as *N*1-cIDPR, and that the hydrogen bonds from the 2′- and 3′-OH on the ribose to the catalytic acid, Glu-226, do not form. The phosphate attached to the *N*1-IMP ribose appears to overlay the crystal structure “southern” ribose phosphate. It may be that the formation of a hydrogen bond between the side chain of Arg-127 and the *N*1-IMP phosphate prevents further penetration of the ligand into the binding site. A key change in the binding of the docked *N*1-IMP ligands is that the hypoxanthine ring binds upside down compared to the crystal structure ligand (*N*1-cIDPR), with the 6 = O pointing down into the binding site, and the *N*3 and *N*9 pointing towards the protein. As previously discussed, when CD38 binds cADPR, the 6-amino substituent forms a hydrogen bond to Glu-146. When *N*1-cIDPR binds to CD38, this hydrogen bond cannot form: there may even be some repulsion between the oxygen lone pairs. Unlike in the cyclic *N*1-cIDPR ligand, the *N*1-IMP ligands have flexibility around the *N*1-ribose bond, allowing the hypoxanthine ring to dock in the most energetically favorable way. The docking into the 2PGJ crystal structure did not allow us to explain the observed differences in binding upon 8-substitution of the *N*1-IMP ligands.

**Figure 13:**
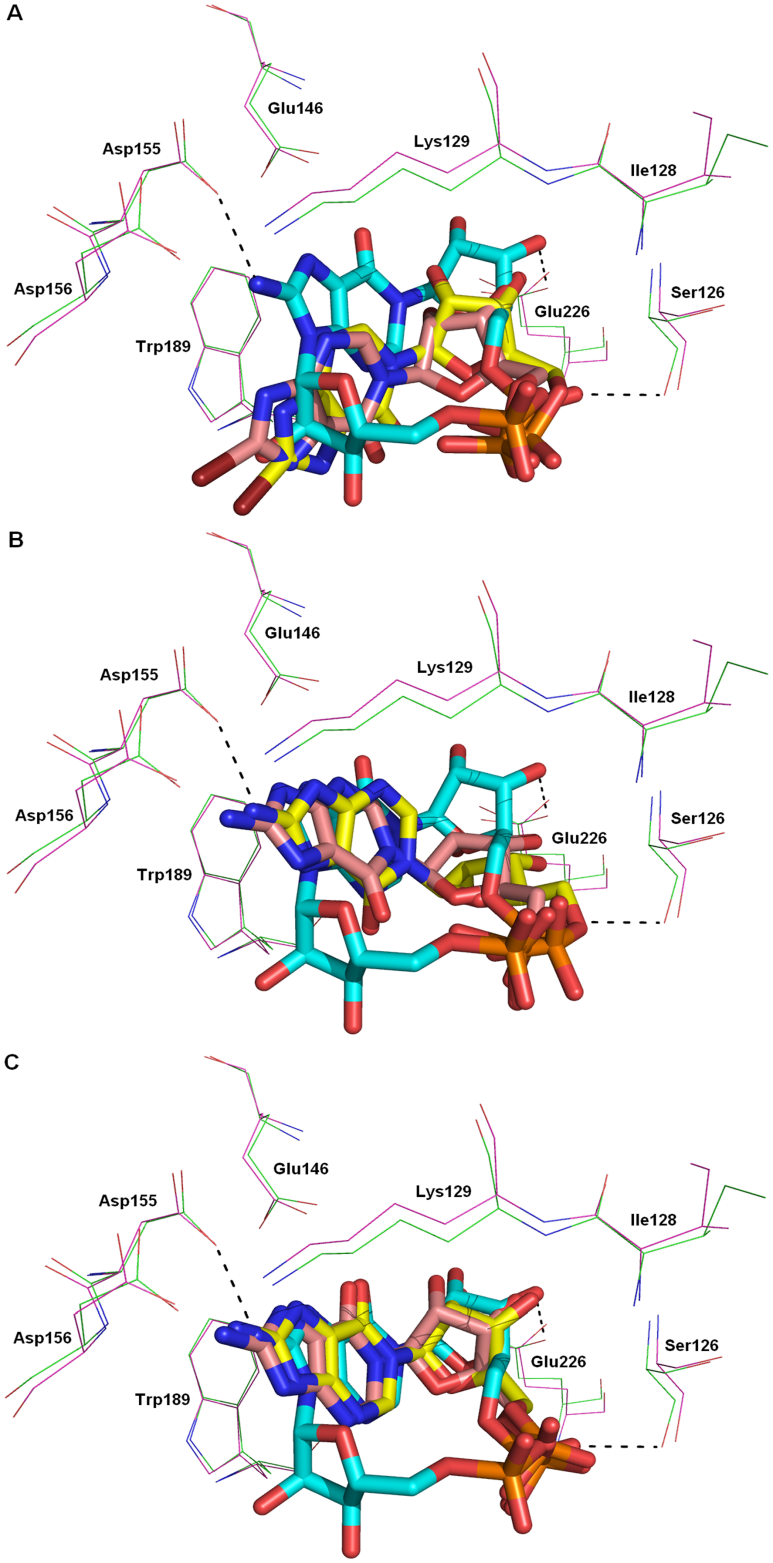
The 3U4H crystal structure with docked ligands. 8-NH_2_-cIDPR docked (protein – green; ligand – cyan), with (A) 8-Br-*N*1-IMP; (B) 8-NH_2_-*N*1-IMP pose 1; (C) 8-NH_2_-*N*1-IMP pose 2. In each case, docked ligand – pink, energy minimized ligand – yellow, protein – purple.

However, when the four ligands were docked into the 3U4H structure, strikingly different poses were adopted by the ligands depending on their 8-substituents. The 8-bromo and 8-iodo *N*1-IMP dock in identical poses which are considerably further out of the binding pocket than the crystallized cyclic compound (Figure 13A). The hypoxanthine ring again docks upside down, with the 6 = O pointing into the binding pocket. Energy minimization of this pose gave identical results for both ligands, pushing the hypoxanthine ring further away from the binding pocket. A shift in the protein structure around the catalytic residue Glu-226 was also observed on minimization, to regenerate the hydrogen bonds to the 2′- and 3′-hydroxyls. The distance of the hypoxanthine ring from the protein precludes any interactions (e.g. between *N*3 or *N*9 and Glu-146) and may explain the poor inhibition of cADPR hydrolysis observed for these two analogues. In contrast, docking the 8-NH_2_-*N*1-IMP ligand into the 3U4H crystal structure gave two contrasting top-ranked results (Figures 13B and C). The same two results were observed upon docking of the *N*1-IMP ligand. In the first pose (Figure 13B), the hypoxanthine ring docks upside down, compared to the cyclic ligand. However with the 8-H or 8-NH_2_ substituents, the hypoxanthine ring overlays the crystal structure ligand very well, suggesting that these two 8-substituents are able to better access the binding pocket. In the second observed pose (Figure 13C), the hypoxanthine ring, “northern” ribose and phosphate all dock in an almost exact overlay of the crystal structure cyclic ligand. Energy minimization of both the docked poses showed minor movement further into the binding pocket, to optimize the interaction between the 2′- and 3′-hydroxyls and Glu-226, and between the phosphate and Ser-126. In both energy minimized poses, the 8-NH_2_ group is able to form a hydrogen bond with Asp-155. Taken together, these docked poses indicate a much better affinity of the *N*1-IMP and 8-NH_2_-*N*1-IMP ligands for the binding site, which agrees with the results observed in the inhibition assay.

This idea was further supported by the results of minimizing the 8-amino *N*1-IMP fragment *in situ*, in both crystal structures (S3). The hydrogen bonds from the 2′- and 3′-OH to the catalytic acid, Glu-226, are preserved as is the hydrogen bond between the phosphate and side chain of Ser-126. The ribose and the 5′-phosphate of the *N*1-IMP fragment and the “northern” ribose and 5″-phosphate of either 8-NH_2_-cIDPR or *N*1-cIDPR lie in almost identical positions. Without the restraint of the macrocycle, the hypoxanthine ring is tilted away from the protein compared to the crystal structure ligand, with the carbonyl oriented away from the oxygens of Glu-146 - this effect is much more pronounced in the 2PGJ crystal structure where *N*1-cIDPR was the original ligand. This tilting may reduce any lone-pair repulsion.

The molecular docking and minimization suggest that the *N*1-IMP fragments, not surprisingly, have flexibility by rotation of the hypoxanthine ring to adopt a better orientation to optimize the interactions between the ligand and the protein for favorable binding. This explains the more efficient binding seen for 8-NH_2_-*N*1-IMP and *N*1-IMP, compared to their cyclic counterparts. Furthermore, the increase in binding seen for 8-NH_2_-*N*1-IMP can be attributed to the formation of the hydrogen bond from the 8-amino substituent to the side chain of Asp155, seen in the energy minimized pose and also observed for 8-NH_2_-cIDPR.

In summary, inhibition of CD38-catalyzed cADPR hydrolysis represents an important approach to the search for agents for intervention in CD38-related diseases, yet this is a relatively unexplored area and there is a need to identify lead structures. Crystallographic studies have identified key residues critical in regulating the multiple functions of the enzyme as well as its catalytic activity. *N*1-cIDPR is the first non-hydrolyzable analogue of cADPR to be crystallized with wild-type CD38; it inhibits cADPR hydrolysis and provides an excellent starting template for structure-based design. Aided by molecular modeling, *N*1-cIDPR was functionalized at the C-8 position of the purine ring. High resolution crystallography and IC_50_ measurements were used to identify 8-amino *N*1-cIDPR as the best cyclic inhibitor of the small series studied, including the unrelated cADPcR with an intact *N*6-amino group. This is thus the first such inhibitor of CD38-catalyzed cADPR hydrolysis evolved using structure-based considerations, and these data suggest the importance of hydrogen bonding interactions both at the C-8 position and via movement of Glu-146 for affinity refinement. Both cADPcR and 8-amino *N*1-cIDPR can reach the catalytic Glu-226 in the active site, mimicking most likely the actual location of cADPR during catalysis and demonstrating the power of our approach to use a structurally engineered non-hydrolyzable analogue with the native enzyme, as opposed to the alternative co-crystallization of cADPR with a mutant enzyme, that was earlier shown to generate a different binding mode. Comparative examination of the crystal structures obtained with cyclic analogues suggested that the 2″- and 3″-hydroxyls and the 5″-monophosphate group are crucial in the inhibition of cADPR hydrolysis, whilst the “southern” ribose monophosphate is apparently not important. This was tested and confirmed using the *N*1-IMP fragments. The lower molecular weight and reduced complexity of these compounds makes them attractive as a starting point for further inhibitor design. The most active inhibitor, 8-NH_2_-*N*1-IMP, is among the best reported non covalent inhibitors of CD38 activity thus far. Further development of these substantially simplified non-cyclic compounds should enable the design of simple, high affinity CD38 inhibitors for pharmacological intervention.

## Supporting Information

Figure S1 **NADase activity of 8-NH_2_-** ***N*** **1-cIDPR.**
(TIF)

Figure S2 **Docked pose of 8-NH_2_-** ***N*** **1-IMP in the 2PGJ crystal structure.** The crystal structure ligand is shown in cyan, and the docked ligand in pink. All four *N*1-IMP ligands docked in an almost identical fashion.
(TIF)

Figure S3 **Energy minimized pose of 8-NH_2_-** ***N*** **1-IMP. (A)** The 3UH4 crystal structure with 8-Amino cIDPR (protein – green; ligand – cyan). The ligand had atoms deleted to generate 8-Amino *N*1-IMP. This protein-ligand complex was put through 1,000 rounds of energy minimization to leave the purple protein and yellow ligand. There is very little difference in the structures. The same hydrogen bonds between the protein and the ligand are formed. **(B)** Energy minimized pose of 8-amino *N*1-IMP in the 2PGJ CD38 crystal structure; Crystal structure protein is shown in green, and the cIDPR ligand in cyan. The minimized protein is shown in purple, and the minimized ligand in yellow.
(TIF)

Figure S4 **Sequence alignment of CD38 from seven species.** Residues identical to those in the human protein are highlighted in yellow. Residues mentioned in the text are shown in blue: several of these residues are shown in Figure 11. Human – *Homo sapiens*, UniProtKB ID P28907. Chimp – *Pan troglodytes*, H2QP89. Monkey – *Macaca fascicularis*, Q5VAN0. Mouse – *Mus musculus*, P56528. Rat – *Rattus norvegicus*, Q64244. Rabbit – *Oryctolagus cuniculus*, Q9MZ03. Cow – *Bos taurus*, Q9TTF5. The sequence alignment was performed by MultAlin, http://multalin.toulouse.inra.fr/multalin/multalin.html.
(TIF)

Table S1 **Crystallographic data and refinement statistics.**
(TIF)
