# Supplementary figures and images for: CD38 Structure-Based Inhibitor Design Using the N1-Cyclic Inosine 5′-Diphosphate Ribose Template
Source: PLoS One. 2013 Jun 19;8(6):e66247. doi: 10.1371/journal.pone.0066247 (PMC3686795; doi:10.1371/journal.pone.0066247)

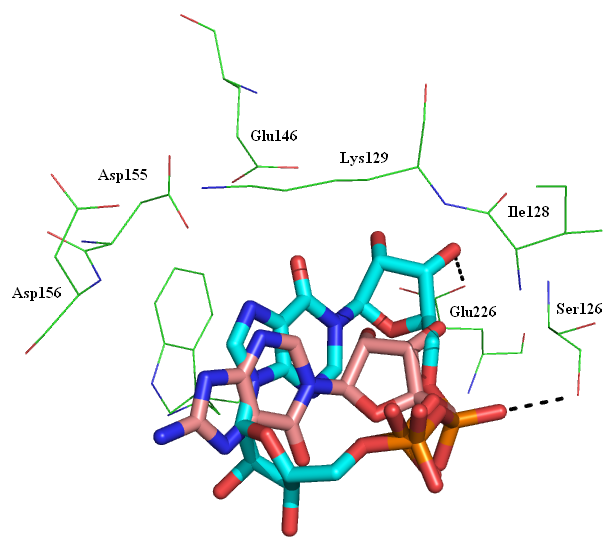

Supplement: Figure S2 — Docked pose of 8-NH2- N 1-IMP in the 2PGJ crystal structure. The crystal structure ligand is shown in cyan, and the docked ligand in pink. All four N1-IMP ligands docked in an almost identical fashion. (TIF) [file pone.0066247.s002.tif]

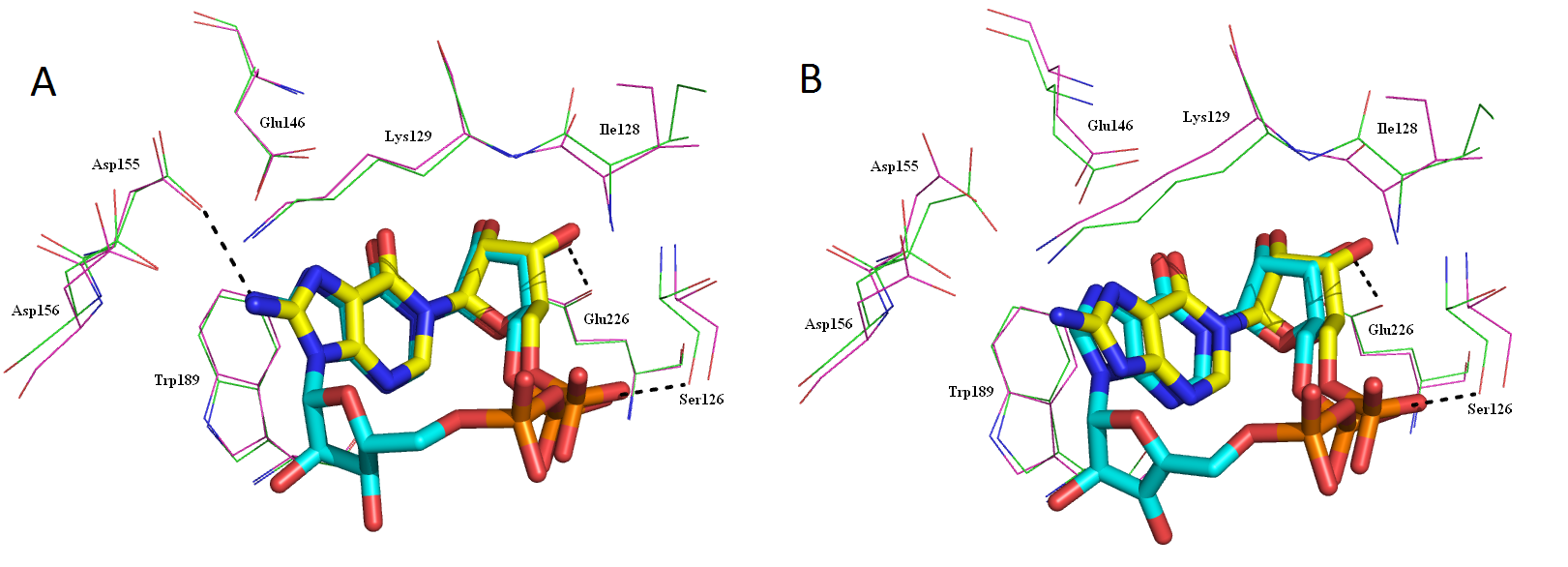

Supplement: Figure S3 — Energy minimized pose of 8-NH2- N 1-IMP. (A) The 3UH4 crystal structure with 8-Amino cIDPR (protein – green; ligand – cyan). The ligand had atoms deleted to generate 8-Amino N1-IMP. This protein-ligand complex was put through 1,000 rounds of energy minimization to leave the purple protein and yellow ligand. There is very little difference in the structures. The same hydrogen bonds between the protein and the ligand are formed. (B) Energy minimized pose of 8-amino N1-IMP in the 2PGJ CD38 crystal structure; Crystal structure protein is shown in green, and the cIDPR ligand in cyan. The minimized protein is shown in purple, and the minimized ligand in yellow. (TIF) [file pone.0066247.s003.tif]

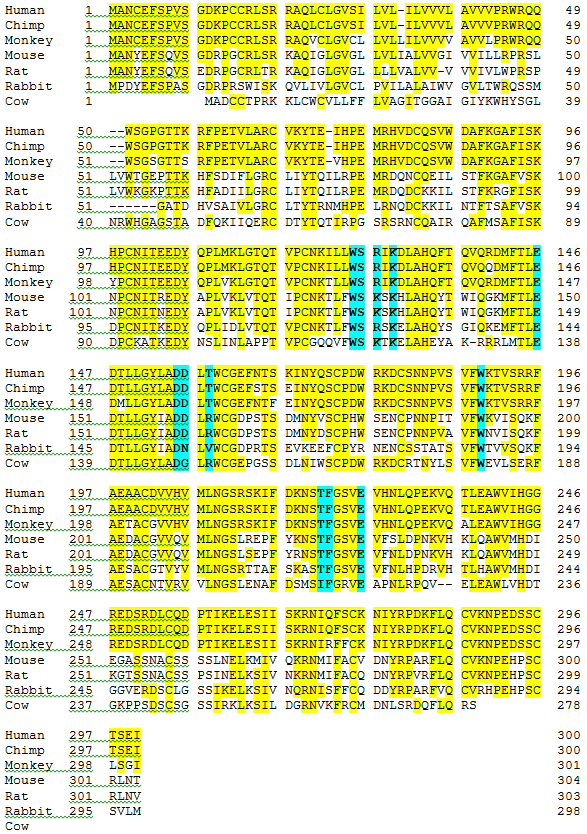

Supplement: Figure S4 — Sequence alignment of CD38 from seven species. Residues identical to those in the human protein are highlighted in yellow. Residues mentioned in the text are shown in blue: several of these residues are shown in Figure 11. Human – Homo sapiens, UniProtKB ID P28907. Chimp – Pan troglodytes, H2QP89. Monkey – Macaca fascicularis, Q5VAN0. Mouse – Mus musculus, P56528. Rat – Rattus norvegicus, Q64244. Rabbit – Oryctolagus cuniculus, Q9MZ03. Cow – Bos taurus, Q9TTF5. The sequence alignment was performed by MultAlin, http://multalin.toulouse.inra.fr/multalin/multalin.html. (TIF) [file pone.0066247.s004.tif]

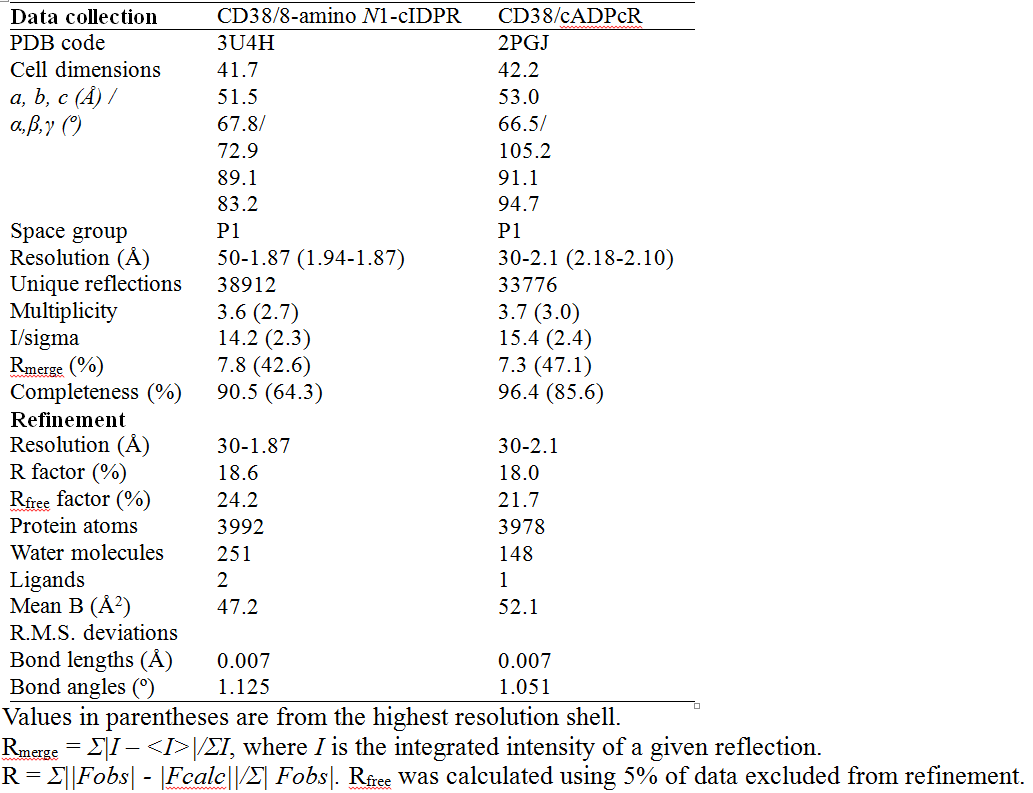

Supplement: Table S1 — Crystallographic data and refinement statistics. (TIF) [file pone.0066247.s005.tif]
